# The Institution Garden

**Published:** 1918-03-23

**Authors:** 


### THE INSTITUTION GARDEN.

### II.—What to Do and How to Do it.

SALADS.

THE next crops to be considered are the salads—lettuce, endive, celery, watercress, corn salad, mustard and cress, and amongst these spinach may be included, for though it is not eaten raw as other salads are, its cultivation is much the same. None of these crops has much value as a quantitative food, but as stores of vitamines they are of the utmost value, and they also have considerable use as aperients, and as affording that bulk and stomach-filling volume which is not without importance in diet. As they are eaten raw, their whole content of salts and vitamines is utilised, and they have this advantage in the garden, that they can be grown as catch-crops. They occupy the ground for a very brief period, and what ground they do occupy could not at the time be put to any other purpose, and would be temporarily wasted; for they may be grown between the rows of crops of longer duration in coming to maturity, such as potatoes, peas, cabbage, and so forth. Salads should therefore be largely grown in every well-managed garden.

RELISHES.

The relishes include herbs in variety, and the onion tribe, including garlic and shallots. Leeks, although botanically closely allied to onions, are from a culinary and horticultural point of view widely separated from them. Leeks rank as green vegetables. They are grown as a substantial crop, and eaten cooked for the sake of their food value, which is low, and not as a relish. With the onion it is otherwise. They are eaten mainly for their flavour, and as a relish and an appetiser, mingled with other viands; but besides this use they have a considerable food value of their own. In this respect they exceed the carrot and the turnip, and almost equal the parsnip; and as they yield a considerable weight per rod of ground, they are profitable from every point of view, and may well occupy at least 12 per cent. of the area of the garden. Shallots are as easy as possible to cultivate, and as they come into use just between the seasons, when last year's onion crop is nearly or quite exhausted, and the onions of the current year are not yet ripe, a breadth should certainly be allowed for shallots. They may have 1 or 2 per cent. of the garden allowed to them.

No garden is worthy to be called a garden that does not contain its plot of herbs, duly cultivated and cared for. We no longer grow the great variety of fragrant and savoury herbs that our ancestors delighted in. Some of them, such as dill, coriander, and anise, are gone out of use; and others, such as lavender, caraway, and wormwood, are grown on so large a scale by growers who specialise in them and grow nothing else, that they no longer find a place in the private herb garden. No one, however, can dispense with mint, sage, thyme, marjoram, and a few other herbs, of which chives must form one, to be used in early salads in place of the spring onions that are not yet to be had. The herb garden occupies, however, so small a space, and herbs are for the most part so permanent a crop, that they may go in with the permanent crops, and need not be considered in allotting space to the crops of the year.

FRUITS.

The last garden crops to be considered are the fruits, ordinarily so-called, including rhubarb, which though, of course, not botanically a fruit, is yet a fruit for culinary and dietetic purposes. The value of fruits as quantitative food is low. They contain on an average less than 1 per cent. of protein, and of carbohydrates from the miserable 2½ per cent. in rhubarb to as much as 17 per cent. in blackberries. Raspberries, gooseberries, and currants average about 12. The value of fruits as quantitative food is therefore low, but they have their value as relishes, as a source of vitamines, as aperients, and as affording a pleasant variety in our food. As they are for the most part permanent or quasi-permanent crops, occupying the same ground for several years, the allotment of space to them forms no part of the regular planning of the gardener at this season of the year; it is usually determined once for all when the garden is laid out. Those who are now plotting out the occupation of newly broken ground may bear in mind that of ground devoted to small fruits, that allotted to gooseberries yields the most food rod for rod, and that allotted to rhubarb yields the least. The only quasi-temporary fruit crop is that of strawberries. They have the very lowest food value of any vegetable crop, and therefore, in spite of their attractive flavour, we must harden our hearts this year and be content with what ground we have already occupied by them, without renewing, as is customary, the three-year-old plot. Grub up the three-year-olds, if you please, but postpone replacing them with young plants till next year.

BEST VARIETIES TO GROW.

This is a matter to which the seedsmen's catalogues give much attention and devote much space, but the information they give is not altogether trustworthy. They give immense prominence to novelties, which, whatever their merits, seldom succeed in the hands of amateurs, and they do not give the kind of information that the amateur wants with regard to the quality of their seeds. Take, for instance, the homely parsnip. There are three varieties of this in common cultivation, and every seedsman offers them all, but no seedsman, as far as appears, indicates their comparative merits or tells the amateur that the old hollow-crowned is the most reliable, but the rankest in flavour; that the Maltese grows to an enormous size, but is comparatively flavourless and apt to go woody at the core; and that the Student is the most toothsome, but yields only a moderate crop; nor does any seedsman tell the amateur that the hollow-crowned parsnip loses its rankness when grown in a light soil, and that in the same circumstance the other varieties are almost devoid of flavour.

Of carrots, the long varieties, such as the old red Surrey, give the largest yield in actual weight, but there is much waste in preparing them for the pot, and the core is apt to grow woody when they are kept long. The horn carrots are rapid in growth and come into use early, but they are small and unsuitable for the main crop, which should consist of the intermediate varieties, which can now be grown to a great size without becoming rank in flavour, and to which there is little waste in cooking.

Beet is easy to grow, and is a fairly nutritious vegetable, containing a good deal of sugar; and in these days of sugar shortage a breadth should be allotted to the sugar beet, which is very easy to grow, from which any intelligent cook can prepare very easily a syrup; which can be made into jam with its own sugar, and will form,

☞ The First Article appeared March 16, p. 513.

**THE INSTITUTION GARDEN—(*continued*).**

after most of the sugar is extracted by boiling and made into syrup, a table vegetable quite as good as its red cousin. The red beet is rarely seen at table except cold and cut into slices. It makes, however, an excellent vegetable served hot with white sauce, or mashed.

Turnips have many enemies, chief among them being drought and the turnip-fly. If they can be grown, the early Milan and the six-weeks' turnip come to table from seed more quickly than any other crop; but the crop is precarious, and will often fail.

Besides these, which form the regulation root crops, and which, with the exception of the turnip, are easy to grow, and scarcely ever fail, there are other root crops worth cultivation for the table. The garden swede is not at all to be despised. It requires longer cooking than other roots, but when well cooked is a palatable vegetable, and affords an agreeable change. Another root to which a row or two may be allotted is salsify. It is not a heavy cropper, but it has a delicate and agreeable flavour, unlike that of any other root. It is called the vegetable oyster, and is perhaps as much like oyster as it is like salmon or a red herring, but scarcely more. Its great drawback is that unless skilfully cooked it comes to table either black, or an ugly mud colour, and is then very unappetising in appearance. The secret of sending it up to table snow-white, as it should be sent, is known to few or no English cooks, is given in no cookery book, and is jealously guarded by the French cooks, who cook it to perfection. It is here given in print for perhaps the first time. Every cook knows that it should be cooked in acidulated water, but few know that it should be prepared, that is, scraped or scrubbed also in water acidulated with lemon juice or vinegar, and fewer still know the crucial secret—that it should be kept under water all the time it is being scraped, and never exposed to the air except for the moment when it is popped into the pot. Scorzonera and the large Spanish radish, as big as a beetroot, and black as a truffle, are other roots that may be cultivated for a change. The girasole, or Jerusalem artichoke, is the least nutritious of all the roots. It has a pleasant flavour, but a very low food value, and though easy of digestion when well cooked, it is very apt to produce flatulence if the cooking is not very thorough, and with some people even if it is.

We have already deprecated the devotion of as much ground as is usual to the cabbage. In old times, when vegetables were few, when the potato was unknown, and so were the very numerous other vegetables that have been introduced from abroad or bred at home, the cabbage was the main stand-by. It was the chief vegetable in the garden, and naturally, therefore, the greatest space of garden ground was allotted to it. Gardeners are a conservative race, and so, indeed, are their masters and mistresses; and though there are now twenty alternative vegetables for every one that existed when the cabbage formed the main vegetable crop, it still, in one form or another, occupies most space in the garden. Its merits and demerits have already been examined, and all that is necessary to add here is that, wasteful as most brassicas are of the ground they occupy, the solid-hearted forms, such as drumhead, beef-heart, and savoy, are the least so, and Brussels sprouts the most. This last brassica yields so little food in comparison with the ground it occupies that, except to preserve the race until more peaceful times, it should scarcely be grown at all.

Much the same may be said of vegetable marrows, pumpkins, and cucumbers. They occupy an inordinate space of ground. Cucumbers need considerable attention and care in cultivation. They are all hungry crops that exhaust the ground; their yield per rod is small, and the food value of what they do produce is very low. A few may be cultivated for an occasional change of diet, but to occupy much ground or much time upon them is ground and time wasted.

There is but one thing more to be said about garden produce, and that is to be said not to the gardener, but to the cook; but the gardener and the cook should work hand in hand. The remaining caution is a very important one: it is that all vegetables should be thoroughly cooked. Well-cooked vegetables are extremely easy of digestion by every well-conducted stomach; ill-cooked vegetables are extremely difficult of digestion by even the best-ordered stomach. Not a little of the troublesome insomnia of elderly people is due to the insufficient cooking of the vegetables that formed part of the evening meal.

